# Beyond the Bench: Prevention Pays for Farmworkers

**DOI:** 10.1289/ehp.115-a28

**Published:** 2007-01

**Authors:** Tanya Tillett

Many agricultural growers use pesticides to protect crops and ensure satisfactory yields. Although the United States has some of the strictest laws governing pesticide applications and re-entry into fields after application, a number of studies have demonstrated that farmworkers are still being exposed to pesticides in the fields and that they may take the chemicals home on their skin and clothing, potentially exposing their families. This risk prompted the University of California, Berkeley, Center for Children’s Environmental Health Research to join with community partners Clinica de Salud del Valle de Salinas, the California Rural Legal Assistance Program, and the Central Coast Grower–Shipper Association to conduct a study on ways to alleviate this hazard. The community-based participatory research study employed a field-based technical intervention designed to reduce farmworker exposure to pesticides and the potential for take-home pesticide exposures.

The partnership, through its Center for Health Assessment of Mothers and Children of Salinas (CHAMACOS), recruited 130 study participants working at two Monterey County strawberry farms during the summer of 2003. Then they initiated preventive measures to decrease direct exposure to malathion (a pesticide commonly used in strawberry fields) and lessen the amount of pesticide residues workers carried home on clothing, skin, and shoes.

“The agricultural work environment is very complex, with changes in weather parameters, soil moisture, crops, tasks, and other factors occurring over short time frames,” says Asa Bradman, CHAMACOS associate director. “We focused on strawberry harvesting to ensure that the crop and task could be held constant. We chose to study malathion because it can be [simultaneously] measured in . . . foliar residue samples, in personal samples such as hand rinse and clothing patch samples, and in urine as the metabolite malathion dicarboxylic acid.”

The study began with surveys, urine sampling, and foliage sampling during a pre-intervention process to determine the farmworkers’ initial exposure levels. Intervention activities began a month later. The team provided protective clothing (lightweight coveralls and disposable gloves), educational sessions at the worksite on exposure prevention, shoe boxes and laundry bags to store work clothing and shoes, and a field station with warm water and soap for handwashing. Post-intervention data collection in October of the same year included surveys, urine sampling, hand rinse sampling, clothing patch sampling, and foliage sampling.

The initial feedback from the participants indicated that the intervention strategies could lower overall exposure if used on a regular basis. One of the solutions, the use of a hot water heater in the field to provide warm water for handwashing, had never been tried before. During the pre-intervention phase, many farmworkers revealed that they had previously avoided washing their hands while picking strawberries because they believed that washing with cold water, which was all that was available, could cause arthritis. The availability of the warm water led to more handwashing and thus less pesticide residue on hands.

The protective clothing and storage for work clothes and shoes also proved helpful to the workers in limiting pesticide exposure. Alicia Salvatore, CHAMACOS intervention study coordinator, says, “Many [farmworkers] reported that they experienced fewer dermal rashes when they used gloves, and almost all workers noticed a marked change in amount of dirt that they carried home at the end of the day on the clothing that they wore under their coveralls. Almost all workers who participated were interested in continuing these behaviors.”

Brenda Eskenazi, CHAMACOS director and principal investigator of the project, sees a necessity and potential for these types of measures, and says the partnership will continue to advance that cause. “We believe it is important to develop sustainable interventions—that is, interventions that are cheap and effective in reducing exposure to pesticides,” she says. “This intervention . . . is an important first step in this direction.”

Bradman says the growers who participated in the intervention were supportive of the project and plan to continue to use the preventive measures to the fullest extent possible. He adds that the community partners in CHAMACOS are presenting their findings to other growers and agricultural organizations, as well as preparing reports for publication.

## Figures and Tables

**Figure:**
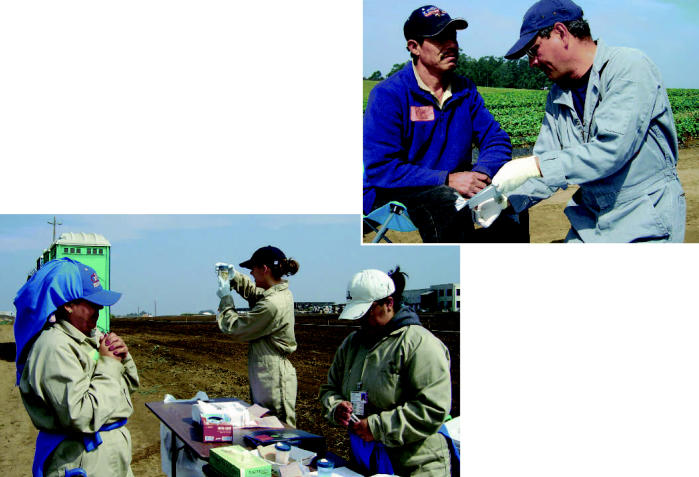
Field work. CHAMACOS researchers collected samples in the field including clothing patches (top) and urine samples (above) to assess pesticide exposures of agricultural workers.

